# Energy and Fatigue Predict Gait Speed and Mood Decline: Results From the Health, Aging and Body Composition Study

**DOI:** 10.1093/geroni/igab046.1431

**Published:** 2021-12-17

**Authors:** Rebecca Ehrenkranz, Xiaonan Zhu, Nancy W Glynn, Caterina Rosano

**Affiliations:** 1 University of Pittsburgh Graduate School of Public Health, Pittsburgh, Pennsylvania, United States; 2 University of Pittsburgh, Pittsburgh, Pennsylvania, United States

## Abstract

Older adults may report high energy alongside tiredness or vice versa; little is known about whether discordant self-reported energy (SEL) and tiredness predict trajectories of mood, cognition, or gait speed. SEL (0-10 scale dichotomized at median) and tiredness (present/absent) were obtained in 2,613 older adults (aged 74.6± 2.87 years) and used to create four groups (energized/not tired, low energy/tired, energized/tired, low energy/not tired). Center for Epidemiologic Studies Depression Scale (CES-D) and gait speed were measured over 10 years; mixed effect models compared trajectories in these domains across each group with low energy/tired group as referent. Each group was significantly associated with CES-D and gait. Adjusting for demographics, the high SEL/not tired group showed the least decline in mood (ß = -0.17, p<0.01); the high SEL/tired group showed the least decline in rapid gait (ß = 0.008, p = 0.02). High SEL may indicate resilience for mood and gait speed decline.

